# Morphology and Magnetic Properties of Ferriferous Two-Phase Sodium Borosilicate Glasses

**DOI:** 10.1155/2014/320451

**Published:** 2014-08-05

**Authors:** Alexander Naberezhnov, Nadezda Porechnaya, Viktor Nizhankovskii, Alexey Filimonov, Bernard Nacke

**Affiliations:** ^1^St. Petersburg State Polytechnical University, Polytechnicheskaya 29, St. Petersburg 195251, Russia; ^2^Ioffe Physico-Technical Institute, Polytechnicheskaya 26, St. Petersburg 194021, Russia; ^3^International Laboratory of High Magnetic Fields and Low Temperatures, Gajowicka 95, 53-421 Wroclaw, Poland; ^4^Leibniz University of Hannover, ETP, Wilhelm-Busch-Street, 30167 Hannover, Germany

## Abstract

This contribution is devoted to the study of morphology and magnetic properties of sodium borosilicate glasses with different concentrations (15, 20, and 25 wt.%) of *α*-Fe_2_O_3_ in an initial furnace charge. These glasses were prepared by a melt-quenching method. For all glasses a coexistence of drop-like and two-phase interpenetrative structures is observed. The most part of a drop structure is formed by self-assembling iron oxides particles. All types of glasses demonstrate the magnetic properties and can be used for preparation of porous magnetic matrices with nanometer through dendrite channel structure.

## 1. Introduction

The macroscopic physical properties of ultradispersed substances under confined geometry differ essentially from the properties of the bulk materials and have a real practical importance. Confined geometry has often been created using artificial or natural insulating porous matrices. The average diameter and topology of pore and wetting ability of embedded materials determine the sizes and spatial organization of embedded substances. But usually the porous matrix plays a passive role in formation of new properties of embedded material: it forms the confined geometry only and does not participate practically (except interface) in modification of macroscopic physical properties of nanostructured materials. On the other hand the utilization of active matrices with magnetic or ferroelectric properties makes it possible to produce a new type of nanocomposite material (NCM) with different spatially separated, but interacted, order parameters. These NCMs with coexisting magnetic and ferroelectric orderings (especially at room temperature) are very perspective materials for practical application and can be considered model objects for nanoferronics [1, 2]. The production of these new multifunctional NCMs is the global idea of our studies. As a magnetic matrix we are going to use the so-called magnetic porous glass obtained by chemical etching of two-phase sodium borosilicate glass doped by hematite (*α*-Fe_2_O_3_). Here it is necessary to note that in this case the interface between both subsystems (ferroelectric and magnetic) can be very developed to support an observable interaction. We have studied the dielectric properties and crystal structure of two-phase alkali borosilicate glasses with different weight concentration of hematite in the initial mixture, that is, 25%: Fe25, 20%: Fe20, and 15%: Fe15 [3, 4], and have shown that during the preparation of these matrices nanoparticles of magnetite (Fe_3_O_4_) and the phase *β*-Fe_2_O_3_ are formed from the hematite *α*-Fe_2_O_3_ added to the initial mixture. Analysis of X-ray high-resolution diffraction (XRD) data [3] has shown that the diffraction sizes of magnetite particles nonlinearly depend on the concentration of hematite in the initial melt: 161 ± 9 Å in Fe15, 150 ± 5 Å in Fe20, and 454 ± 6 Å in Fe25. From phase analysis of diffraction patterns we have determined that Fe15 glasses contain 77 ± 6 wt.% and 23 ± 6 wt.% *β*-Fe_2_O_3_ of total iron oxides concentration. In Fe25 glasses the admixture of *β*-Fe_2_O_3_ has not exceeded 2 wt.% and in Fe20 glasses we have not observed any traces of *β*-Fe_2_O_3_ phase. The diffraction size of *β*-Fe_2_O_3_ nanoparticles within Fe15 glasses was 208 ± 10 Å [3]. Magnetite (Fe_3_O_4_) is the ferrimagnetic materials and we can expect the appearance of magnetic properties for these glasses, but for production of porous magnetic glasses it is necessary to have the two-phase structure of glasses consisting of interpenetrative silica skeleton and chemically unstable phase, which can be removed at chemical etching of two-phase glasses [5]. The formation of this two-phase structure has been produced by the special heat treatment of alkali borosilicate glasses on cooling [5, 6]. Therefore on the next stage we have studied the morphology and magnetic properties of these ferriferous alkali borosilicate glasses and their convenience for preparation of magnetic porous glasses (MPG). It is necessary to note that MPG are very interesting not only for preparation of multifunctional (ferroelectric and magnetic) nanocomposite materials but for various other applications as well such as biocompatible matrices for drag delivery [7, 8], for hyperthermia treatment of cancer [9], for tissue engineering and regeneration [10, 11], and for immobilization of enzymes [12–14]. These matrices have potential application in spin electronic devices, high density magnetic data storage, and magnetic random access memory [15] on the basis of exchange bias effect [16].

## 2. Methods and Measurement Procedures

The ferriferous borosilicate glasses were produced in Grebenshchikov Institute of Silicate Chemistry (Russian Academy of Sciences) and were kindly given for study of their morphology and magnetic properties. After melting the produced glasses have been kept up at 550°C during 120 hours for phase separation. As a result of this process one can produce two-phase glasses in which the channels of chemically unstable phase have penetrated into a silica skeleton, but formerly this technology was used for production of conventional (nonmagnetic) porous alkali-borosilicate glasses [5, 6]. After cooling down to RT the glasses were cut and all samples were rectangular polished plates 10 × 10 × 2 mm^3^. The morphology and surface magnetic properties were measured using the atomic-force microscope (AFM) Attotube AFM1 including magnetic force microscopy (MFM) mode. Attotube AFM1 can operate in the temperature diapason 4–285 K and at applied magnetic field up to 9 T. At studies of surface topology we used the semicontact method and silicon needles with a curvature radius of 10 nm. In the MCM mode the silicon needles with 60 nm cobalt coating (coercive force 20–30 mT) and 20 nm chromium protective layer were used. In these measurements the total curvature radius was 90 nm for magnetic needles. For generation of MFM images we utilized two-pass method: at the first pass the surface topology was obtained (in semicontact mode); at the second pass the needle was lifted up and followed the trajectory obtained at the first pass. The distance “needle-sample” was kept at 100 nm during scanning and we measured the magnetic response only. So additionally to study of surface topology the images of surface magnetization distribution in the zero applied fields have been obtained. In applied magnetic fields the measurement procedure was the same. The images of inner glass structure have been studied by transmission electron microscopy (TEM) using replicas from the fresh chip. We have studied the surface magnetic response of these glasses using magnetic force microscopy at applied fields (−)10^3^ Oe–4 ∗ 10^3^ Oe at 4.2 K; the field dependencies of ferriferous glasses magnetization were measured at 4.2 K and in the diapason* H* (−)1.4 ∗ 10^5^–(+)1.4 ∗ 10^5^ Oe in International Laboratory of High Magnetic Fields and Low Temperatures (Wroclaw, Poland) using vibration magnetometer and superconducting magnet of Oxford Instrument.

## 3. Results and Discussion

### 3.1. Morphology

**Figure 1:**
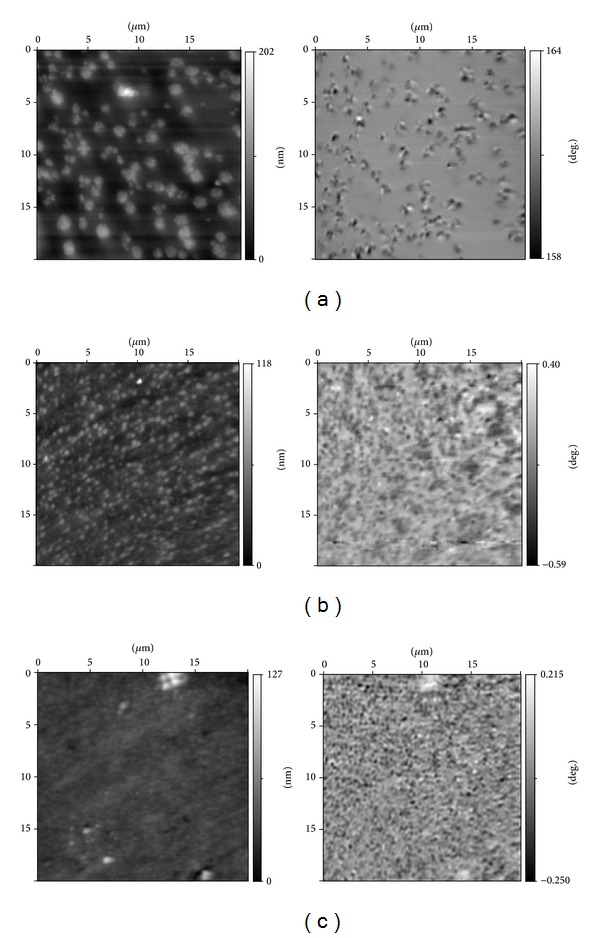
Topography (left) and magnetic response (right) of the ferriferous two-phase glasses Fe25 (a), Fe20 (b), and Fe15 (c); *H* = 0 Oe and *T* = 300 K.

In Figure 1 the topography (left) and the magnetic response (right) at zero applied field and at room temperature for the Fe15, Fe20, and Fe25 are presented. In all topology images one can see the large agglomerates upthrusted from common background (right scale in nanometers on the left figures). A similar picture is characterized for two-phase glasses with drop structure [5, 6, 17]. The average size of these agglomerates increases at a growth of iron oxide concentration. We have estimated the average lateral sizes (230 ± 10 nm for Fe15, 450 ± 10 nm for Fe20, and 940 ± 10 nm and 500 ± 10 nm for Fe25) and average heights over a common background of agglomerates: 9 ± 2 nm, 27 ± 4 nm, and 57 ± 6 nm for the Fe15, Fe20, and Fe25, respectively. The drop structure is not suitable for production of porous glasses. It is necessary to have the glasses with two interconnected (or interpenetrated) phases: the first one is a so-called chemically unstable alkali-rich borate phase soluble in the hot acids; the second one is practically pure silica skeleton. The procedure of porous glasses preparation is described, for example, in the review [5, 6].

**Figure 2:**
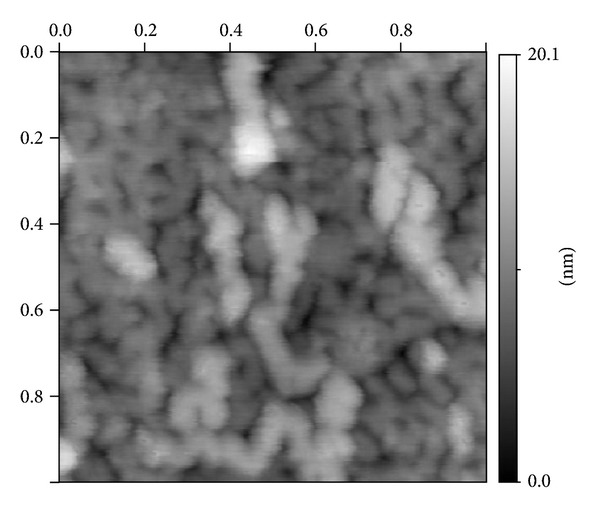
Surface of two-phase ferriferous alkali borosilicate glass Fe25 in a part free from agglomerates. The snapshot size - 1 × 1 *µ*m^2^. The scale on the right-relief height over average background.

**Figure 3:**
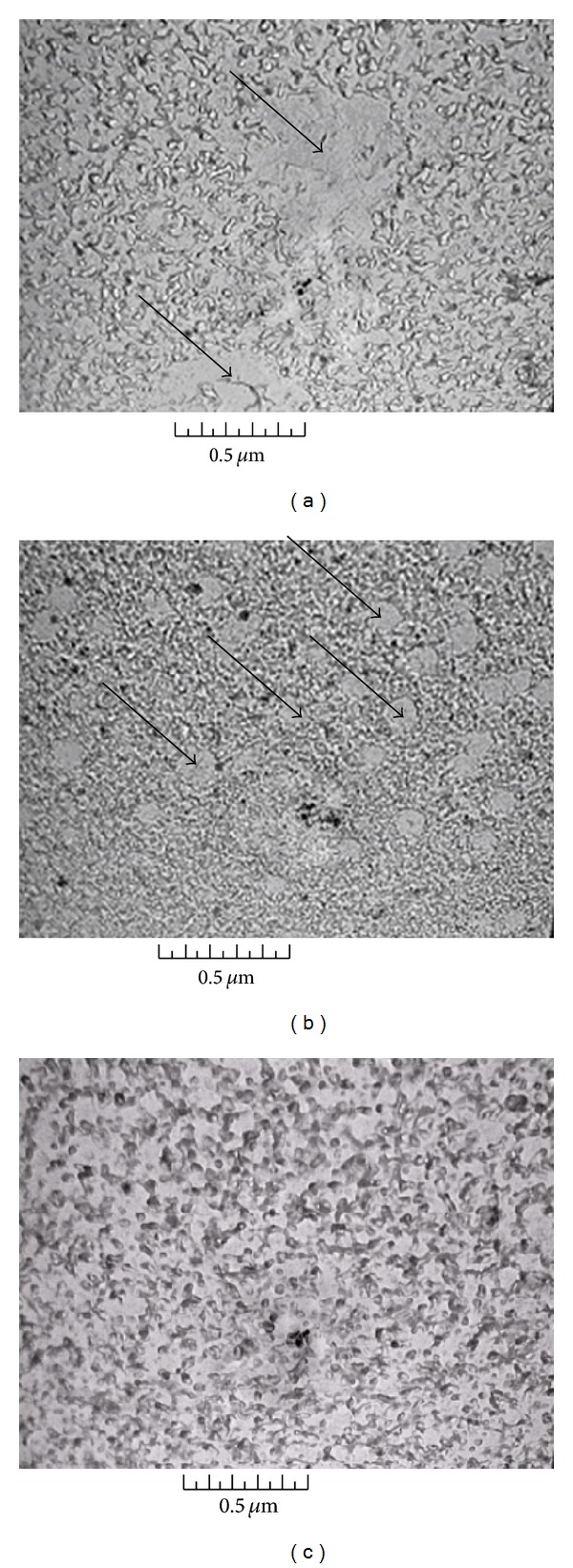
TEM data for the Fe25 (a), Fe20 (b), and Fe15 (c) glasses. Arrows indicate the agglomerates formed the drop structure [18].

In Figure 2 the part of ACM image (1 × 1 *μ*m^2^) for the Fe25 glass surface, where there is no agglomerates, is presented and one can see that the typical dendrite (for two interpenetrated phases) structure exists there. AFM results are confirmed by TEM data [18, 19] presented on Figures 3(a), 3(b), and 3(c). The arrows in Figures 3(a) and 3(b) indicate the positions of large agglomerates formed in the drop structure. Simultaneously as it is easy to see in Figures 3(a) and 3(b) two interconnected phases are evidently present at samples. So we can believe that in these glasses there is a coexistence of drop phase and two interconnected (interpenetrated) phases and it is possible to use these materials for porous glass production.

### 3.2. Magnetic Properties

The distributions of magnetic response on surfaces of the Fe25, Fe20, and Fe15 glasses at zero applied magnetic field and at room temperature are presented in Figure 1, right column. The small dark and white regions corresponding to the different (resp., magnetization of magnetic cantilever) directions of magnetization are evidently observed. The positions of these magnetic anomalies coincide with locations of surface agglomerates, at which the several nearest magnetic anomalies with different magnetization direction correspond to common surface agglomerate. It means that these agglomerates have the magnetic properties and contain a magnetite really because Fe_3_O_4_ at room temperature demonstrates ferrimagnetic properties.

**Figure 4:**
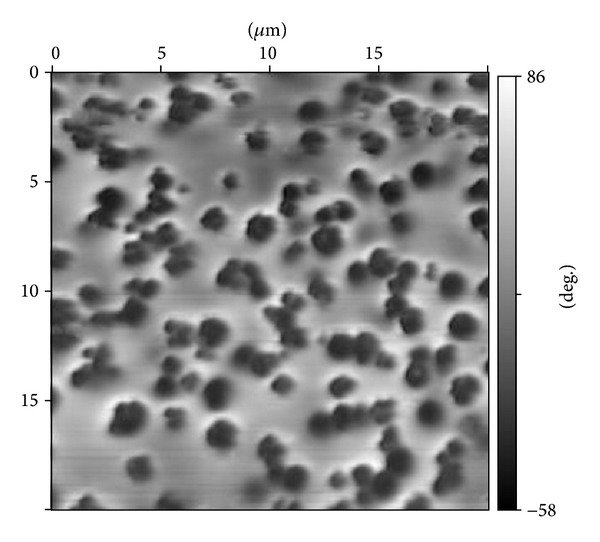
MFM image of the Fe25 glass surface at 4.2 K and at *H* = 4000 Oe. The scanned region - 20 × 20 *µ*m^2^. Scale (in degrees) on the right value of phase at measurement of magnetic response in MFM mode.

MFM image in Figure 4 presents the distribution of surface magnetization for the Fe25 glass at 4.2 K and at *H* = 4000 Oe. It is easy to see that at these conditions one monochromatic (dark) magnetic region corresponds to one surface agglomerate; that is, at *H* = 4000 Oe the magnetization of every magnetic anomaly orients itself along the applied magnetic field and every surface agglomerate becomes a magnetic monodomain. In case of the Fe25 glass the average lateral size of this monodomain is equal to 980 nm and practically agrees with the lateral size of agglomerates obtained from surface topology studies taking into account experimental uncertainties and resolution.

**Figure 5:**
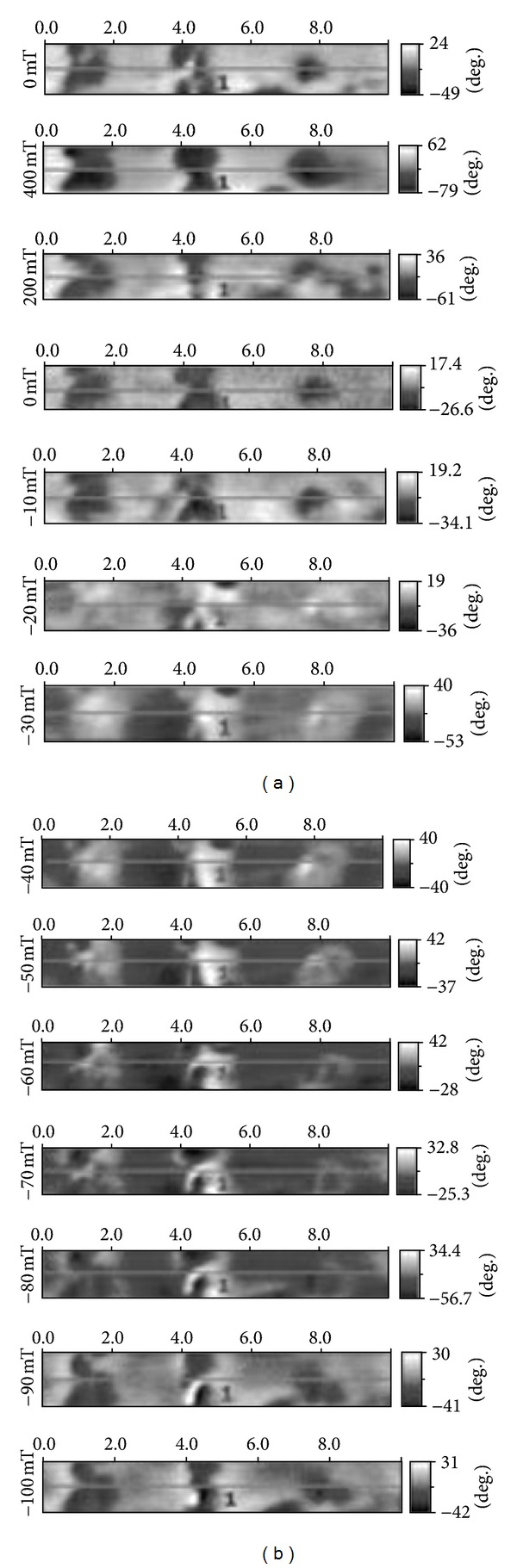
MFM images of magnetization switching of three neighbor agglomerates on surface of the Fe25 glass. The horizontal scales are in *μ*m. The vertical scales (in degrees) values of phase at measurement of magnetic response in MFM mode.

To study of magnetic switching at 4.2 K we have chosen three neighbor agglomerates on surface of the Fe25 glass and have recorded MCM images at variation of magnetic field induction in the diapason 4–(−) 1 ∗ 10^3^ Oe (Figure 5). Initially the directions of magnetization of agglomerates and magnetic cantilever have coincided (left column from top to down) and the three dark areas have been present at the images. In diapason (−) 1–(−) 3 ∗ 10^2^ mT we have observed the change of agglomerates color from dark to white—this process connects with the switching of magnetic cantilever. At subsequent increase of magnetic field these areas continue to change color fluently and at 750–900 Oe become dark finally. One can consider this value (750–900 Oe) to be an estimation of coercive force for the Fe25 glass.

**Figure 6:**
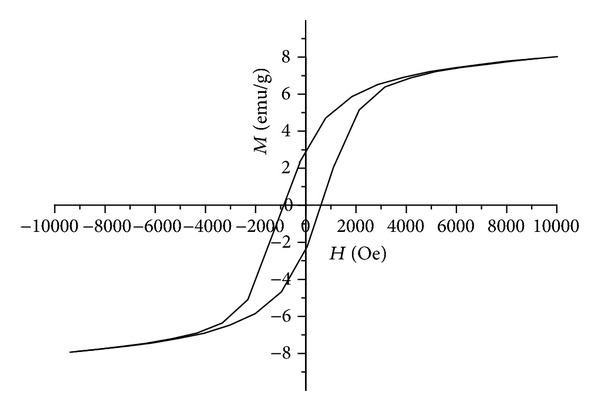
Magnetization of the Fe20 glass versus applied magnetic field at 4.2 K.

The field dependence of the Fe20 glass magnetization has been measured at 4.2 K by vibration magnetometer and is presented in Figure 6. It is easy to see the presence of the hysteresis loop and a saturation of magnetization at fields above 10000 Oe. The same field dependence is typical for superparamagnetic system, in particular for Stoner-Wohlfarth ensemble [20] of monodomain anisotropic magnetic particles. The coercive field of hysteresis loop is about 870 Oe and is in a good agreement with the value of* H*_*C*_ obtained for the Fe25 glass from the AFM data. Here it is necessary to note that these results differ essentially from the results presented in the paper [21] for 20Na_2_O - 1.0Al_2_O_3_ - 13B_2_O_3_ - 6.3CaO - 0.2Sb_2_O_3_ - 4.5BaO - (55−*x*)SiO_2_ - (*x*)Fe_2_O_3_ (where* x* = 0, 5, 10, 15, 20, and 25) glasses where the hysteresis loop has been observed at Fe_2_O_3_ concentration of 25% only. For other concentrations (5–20%) these glasses have demonstrated a paramagnetic behavior.

So the combined analysis of X-rays diffraction [3], MFM, and magnetic properties data permits concluding that in ferriferous alkali borosilicate glasses during the melt and phase separation processes the self-organization of iron oxides nanoparticles into large agglomerates with magnetic properties takes place.

## 4. Conclusion

In the Fe15, Fe20, and Fe25 ferriferous alkali borosilicate glasses the drop-like and two-phase interpenetrative structures coexist and it is possible to use these glasses for production of porous glasses with magnetic properties. At preparation of glasses the transition of initial hematite (*α*-Fe_2_O_3_) into magnetite (Fe_3_O_4_) takes place, at which the magnetite nanoparticles with average sizes of 16 nm (for Fe15 and Fe20) and 46 nm (for Fe25) [3] self-assemble in the large agglomerates on surface and inside samples. The average sizes of these agglomerates depend on the initial *α*-Fe_2_O_3_ concentration. All types of these glasses demonstrate the magnetic properties corresponding to Stoner-Wohlfarth ensemble of monodomain anisotropic magnetic particles. The field dependences of magnetization and the values of coercive fields have been obtained. The Fe20 glass does not contain the visible admixture of other iron oxides and is the most suitable for production of magnetic porous glasses and for creation of multiferroic artificial nanocomposite materials with spatial-separated magnetic and ferroelectric orderings with developed interface at ambient conditions.
